# Associations between Polybrominated Diphenyl Ether (PBDE) Flame Retardants, Phenolic Metabolites, and Thyroid Hormones during Pregnancy

**DOI:** 10.1289/ehp.1003235

**Published:** 2011-06-29

**Authors:** Heather M. Stapleton, Sarah Eagle, Rebecca Anthopolos, Amy Wolkin, Marie Lynn Miranda

**Affiliations:** 1 Nicholas School of the Environment, Duke University, Durham, North Carolina, USA; 2 National Center for Environmental Health, Centers for Disease Control and Prevention, Atlanta, Georgia, USA; 3 Department of Pediatrics, Duke University, Durham, North Carolina, USA

**Keywords:** flame retardants, OH-BDEs, PBDEs, pregnancy, thyroid hormones

## Abstract

Background: Polybrominated diphenyl ethers (PBDEs) are chemical additives used as flame retardants in commercial products. PBDEs are bioaccumulative and persistent and have been linked to several adverse health outcomes.

Objectives: This study leverages an ongoing pregnancy cohort to measure PBDEs and PBDE metabolites in serum collected from an understudied population of pregnant women late in their third trimester. A secondary objective was to determine whether the PBDEs or their metabolites were associated with maternal thyroid hormones.

Methods: One hundred forty pregnant women > 34 weeks into their pregnancy were recruited into this study between 2008 and 2010. Blood samples were collected during a routine prenatal clinic visit. Serum was analyzed for a suite of PBDEs, three phenolic metabolites (i.e., containing an –OH moiety), and five thyroid hormones.

Results: PBDEs were detected in all samples and ranged from 3.6 to 694 ng/g lipid. Two hydroxylated BDE congeners (4´-OH-BDE 49 and 6-OH-BDE 47) were detected in > 67% of the samples. BDEs 47, 99, and 100 were significantly and positively associated with free and total thyroxine (T_4_) levels and with total triiodothyronine levels above the normal range. Associations between T_4_ and PBDEs remained after controlling for smoking status, maternal age, race, gestational age, and parity.

Conclusions: PBDEs and OH-BDEs are prevalent in this cohort, and levels are similar to those in the general population. Given their long half-lives, PBDEs may be affecting thyroid regulation throughout pregnancy. Further research is warranted to determine mechanisms through which PBDEs affect thyroid hormone levels in developing fetuses and newborn babies.

Polybrominated diphenyl ethers (PBDEs) are chemical additives applied to numerous types of furniture and electronic and electrical items to meet state and federal flammability standards. Historically, three PBDE commercial mixtures have been used in consumer products (Alaee et al. 2003; de Wit 2002). These three mixtures are referred to as pentaBDE, octaBDE, and decaBDE. The use of pentaBDE and octaBDE has been phased out or banned in the United States and different areas of the world starting in 2002. DecaBDE continues to be used in consumer products, although manufacturers have recently agreed to phase out the use of decaBDE by 2012 (Hess 2009). Despite this phaseout, products containing PBDEs (e.g., furniture, TVs) are still in use in many homes, and exposure will likely continue for some time.

Several studies have now demonstrated that PBDEs are ubiquitous contaminants commonly detected in human tissues (Hale et al. 2003; Sjodin et al. 2003, 2008). The dominant PBDE congeners detected in human tissues are from those found in the pentaBDE mixture, likely due to the greater historical use of pentaBDE in North American furniture relative to other regions.

PBDEs have a chemical structure similar to thyroid hormones, most notably thyroxine (T_4_). In laboratory animal studies, PBDEs affect thyroid regulation by decreasing circulating levels of thyroid hormones, altering expression of genes that encode thyroid-regulating proteins, and reducing activity of thyroid-regulating enzymes. Furthermore, *in vitro* studies have shown that PBDEs and their CYP P450–mediated metabolites, hydroxylated PBDEs, can compete for binding sites on thyroid hormone transporters in serum (Marchesini et al. 2008; Meerts et al. 2000).

PBDE levels in maternal and cord blood tissues have been associated with cryptorchidism in male infants, lower birth weights in babies, and neurodevelopmental and behavior problems in children, including reductions in IQ (Chao et al. 2007; Herbstman et al. 2010; Main et al. 2007; Roze et al. 2009). In adults, PBDE levels have been associated with significant alterations in hormone levels and fecundability in women (Harley et al. 2010; Meeker et al. 2009).

These studies raise concerns about exposure of women to PBDEs during pregnancy and the potential of PBDEs to affect growth and development of the fetus. Thyroid hormones are supplied to the developing fetus via placental transfer during the first half of pregnancy. Late in the second trimester, the fetus begins to produce its own thyroid hormones; however, the fetus still relies on maternal inputs to stabilize the thyroid hormone supply. Thyroid hormone delivery to the fetus is essential for fetal brain development. A recent study (Chevrier et al. 2010) examined PBDE levels and thyroid hormones in serum collected from pregnant women during their second trimester. They observed an inverse association between PBDEs and thyroid-stimulating hormone (TSH) but no relationship with T_4_. The odds of subclinical hyperthyroidism (low TSH but normal T_4_) were significantly higher in individuals with the highest PBDE levels relative to individuals with the lowest PBDE levels.

The goal of this study was to examine PBDE levels in a pregnancy cohort in North Carolina and provide more data on the associations between PBDEs and thyroid hormones. Samples were from an ongoing pregnancy cohort study examining social and environmental risk factors for adverse pregnancy outcomes. To complement the existing literature and to determine whether previously noted associations between PBDEs and thyroid hormone levels observed during the second trimester existed during the third trimester, we examined these relationships in women > 34 weeks pregnant. This study also expands previous research by including measurements of hydroxylated PBDEs, which have been hypothesized, but not previously tested, to have more potent effects on thyroid hormone regulation than PBDEs. In addition, our study focuses primarily on a cohort of African-American women, a population generally understudied in PBDE exposure research.

## Materials and Methods

*Participant recruitment.* Participants were recruited from within an ongoing observational prospective cohort study assessing the joint effect of social, environmental, and host factors on pregnancy outcomes [the Healthy Pregnancy, Healthy Baby (HPHB) Study] (Burgette and Reiter 2010; Miranda et al. 2010; Swamy et al. 2011). The HPHB study enrolls pregnant women from the Duke Obstetrics Clinic and the Durham County Health Department Prenatal Clinic. Women receiving prenatal care at these sites were eligible to participate if they were at least 18 years of age, English literate, between 18 and 28 weeks of gestation at study enrollment, lived in Durham County, were planning on delivering at Duke University Medical Center, and did not have a multiple gestation or any known fetal genetic or congenital abnormalities. All patients > 34 weeks of gestation enrolled in the HPHB study and who were routine patients at the Durham County Health Department’s Prenatal Clinic at the Lincoln Community Health Center (Durham, NC) between September 2008 and June 2010 were eligible to participate in the present study. The choice of this clinic ensured a predominantly African-American study population. Women were approached during their routine third trimester laboratory visit (35–36 weeks) for participation in this study. Of all women approached for this study, > 97% agreed to participate. After women gave informed consent, samples were collected during the third trimester of pregnancy (> 34 weeks of gestation). All aspects of this study were carried out in accordance with a human subjects research protocol approved by the Duke University Institutional Review Board.

*Sample collection.* Consenting women filled out a short questionnaire that documented information regarding their personal, diet, and lifestyle characteristics. Two tubes of blood were collected during the clinic visit: one 4.5-mL tube (BD Vacutainer plasma separation with lithium heparin) and one 10-mL tube (BD Vacutainer serum separator). The tubes were allowed to sit for several hours in a refrigerator and were later centrifuged (within 24 hr) at 3,500 RMP for 5 min to isolate the plasma and serum, respectively. The 10-mL tubes (~ 4–5 g serum) were stored at –20°C until analysis. The smaller tubes were sent to the clinical laboratory at Duke University Hospital for thyroid hormone analysis within 8 hr of centrifugation.

*Thyroid hormone analysis and lipid measurement.* Plasma samples were analyzed by the clinical laboratory at Duke University Hospital (Durham, NC). TSH was measured using the Access HYPERsensitive hTSH assay; free T_4_ (FT_4_) was measured using the Access Free T_4_ assay; total T_4_ (TT_4_) was measured using the Access Total T_4_ assay; free triiodothyronine (FT_3_) was measured using the Access Free T_3_ assay; and total T_3_ (TT_3_) was measured using the Access Total T_3_ assay. All Access assays were paramagnetic particle, chemiluminescent immunoassays manufactured by Beckman Coulter Inc. (Fullerton, CA). Lipid content of the serum was determined using an enzymatic method based on measurements of serum cholesterol and triglycerides (Covaci et al. 2006).

*Chemicals.* Information on the specific internal, recovery, and quantitative standards used can be found in Supplemental Material, p. 2 (http://dx.doi.org/10.1289/ehp.1003235).

*Sample extraction.* Serum samples (approximately 3–5 g) were extracted using a previously published method (Stapleton et al. 2008). More information regarding sample extraction can be found in Supplemental Material, p. 2 (http://dx.doi.org/10.1289/ehp.1003235).

*Sample analysis.* Extracts were analyzed for 27 PBDE congeners using a method reported by Stapleton et al. (2008). A subset of 57 serum extracts was spiked with 10 ng ^13^C 6-OH-BDE 47 and ^13^C α-HBCD, then blown to dryness under pure nitrogen gas. Dried extracts were reconstituted in 100 µL methanol and analyzed using liquid chromatography tandem mass spectrometry (LC/MS-MS) for phenolic metabolites including 2,4,6-tribromophenol (246 TBP), 4´-hydroxy-2,2´,4,5´-tetrabromodiphenyl ether (4´-OH-BDE-49), 6´-hydroxy-2,2´,4,5´-tetrabromodiphenyl ether (6´-OH-BDE-49), 6-hydroxy-2,2´,4,4´-tetrabromodiphenyl ether (6-OH-BDE-47), 6-hydroxy-2,2´,4,4´,5-pentabromodiphenyl ether (6-OH-BDE-99), and alpha-, beta- and gamma hexabromocyclododecane (HBCD). More information on the LC/MS-MS method can be found in Supplemental Material, pp. 2–3 (http://dx.doi.org/10.1289/ehp.1003235).

*Quality control.* Bovine serum (Invitrogen, Carlsbad, CA) and deionized water were both used as laboratory blanks, and SRM 1958 [Fortified Human Serum; NIST (National Institute of Standards and Technology), Gaithersburg, MD] was used for quality assurance to test performance of the extraction method for PBDEs and HBCD. Further details on the calculation of method detection limits (MDLs) and internal standard and SRM recoveries can be found in Supplemental Material (http://dx.doi.org/10.1289/ehp.1003235).

*Statistical analysis.* Statistical analyses were performed using Stata 11 (StataCorp, LP, College Station, TX). Values below MDL were assigned a value equal to half the detection limit for statistical analyses. Summary statistics were computed for thyroid hormones, individual and total PBDEs, and phenolic metabolites, using only those compounds with detection frequencies ≥ 50%. Correlations among PBDEs, metabolites, and thyroid hormones were examined using Spearman rank-order correlation. The distributions of thyroid hormone, PBDEs, and metabolites were assessed for normality (Shapiro–Wilkes) and log-transformed if they were log-normally distributed. We implemented multiple linear regression analysis to assess the association between thyroid hormone levels and PBDE/metabolite levels, controlling for maternal characteristics known to influence thyroid hormone levels, including smoking status (two categories of nonsmoker and ever-smoked before 28 weeks of gestation), race (non-Hispanic black or other), age (three categories: 18–19, 20–24, and 25–39 years), gestational age at blood draw (weeks), and parity (operationalized as whether or not the infant was firstborn). In addition, we used logistic regression (adjusted for the same covariates) to examine whether PBDE or PBDE metabolite levels were associated with TT_3_ levels above the normal range in the general population (80–178 ng/dL) or FT_4_ levels below the normal range (0.52–1.21 ng/dL) and ordered logistic regression to estimate associations of PBDEs/metabolites with low, normal, or high hormone TT_4_ levels (normal range 5.5–10.8 µg/dL). For TSH, the normal range in the general population was defined as 0.34–5.66 µIU/mL. Few observations fell outside the normal range for FT_3_ (2.2–3.8 pg/mL), precluding further analysis. Significance levels were set at *p <* 0.05.

## Results

*Population characteristics.* Between September 2008 and June 2010, 140 pregnant women enrolled in this study. Women were all residents of Durham County, North Carolina. Characteristics of the 137 participants are presented in Table 1. Eighty percent of the women in the study were non-Hispanic black, and 23% were < 20 years of age. Note that oversampling of non-Hispanic blacks is an intentional component of the parent HPHB Study. This was the first pregnancy for roughly half of the women enrolled in this study, and more than 87% completed high school. Of all the women participating in this study, only one reported a personal history with thyroid problems; she was not excluded from this study.

**Table 1:** Cohort characteristics (*n* = 137).

Characteristic	*n* (%)
Maternal race	
Non-Hispanic white	12 (9)
Non-Hispanic black	110 (80)
Hispanic	12 (9)
Other	3 (2)
Maternal age (years)	
18–19	32 (23)
20–24	69 (50)
25–39	36 (26)
Parity	
First birth	70 (51)
Second birth	38 (28)
Third birth	19 (14)
Fourth birth	6 (4)
Five or more	4 (3)
Male infant sex*a*	62 (46)
Maternal education	
Less than high school	19 (14)
High school diploma	72 (53)
More than high school	46 (34)
Not married	127 (93)
Smoking	
Nonsmoker	90 (66)
Smoked before 28 weeks of gestation	47 (34)
**a**Total *n* = 133 because of missing values. Percentages may not sum to 100 because of rounding.	

*Thyroid hormones.* Thyroid hormone data were available for only 137 of the 140 women enrolled (Table 2); however, we had a total of 136 thyroid hormone measurements per individual hormone type because of problems with the laboratory measurement of some hormones in a few samples. TSH, FT_4_, and FT_3_ were log-normally distributed, whereas TT_4_ was normally distributed. TT_3_ was not normal or log-normally distributed and was log-transformed for regression analysis. With the exception of one participant with a TSH level of 0.31 µIU/mL, all serum samples were found to have TSH levels within normal ranges. Pregnancy can affect thyroid hormone levels, and normal ranges presented here are for the general population. Approximately 10% of the serum samples (*n* = 13) had FT_4_ levels below normal ranges, and one was just above normal at 1.22 ng/dL. For measurement of TT_4_, 28% of the serum samples had levels below the normal range, and five samples had TT_4_ levels above normal. Five samples had FT_3_ levels below the normal range, and two were higher than normal. In contrast, approximately 63% of the serum samples displayed TT_3_ levels higher than normal, whereas the remaining 37% were within normal ranges.

**Table 2:** Thyroid hormone levels and PBDE and metabolite concentrations (nanograms per gram lipid) measured in serum from pregnant women.

Detection frequency (%)	Geometric mean (95% CI)	Percentile						
		Variable	MDL	Min	Max	25th	50th	75th	95th
Thyroid hormones (*n* = 136)																		
TSH (µIU/mL)				100.00		1.25 (1.15–1.37)		0.31		5.38		0.89		1.28		1.76		2.91
TT_4_ (µg/dL)				98.53		6.1 (5.4–6.7)		0.3		11.9		5.2		6.9		8.5		10.4
FT_4_ (ng/dL)				100.00		0.67 (0.64–0.69)		0.40		1.22		0.59		0.67		0.75		0.89
TT_3_ (ng/dL)				100.00		195 (186–204)		100		411		165		188		221		349
FT_3_ (pg/mL)				100.00		2.71 (2.65–2.77)		1.85		4.25		2.47		2.71		2.97		3.39
PBDEs (*n* = 137)																		
BDE-28		1.2–3.0		38.69		NA		< 1.2		16.89		0.60		0.60		2.58		6.00
BDE-47		2.0–4.5		94.89		16.5 (13.64–19.98)		< 2.0		297.45		8.98		18.87		30.64		114.36
BDE-66		1.2		2.19		NA		< 1.2		3.93		0.60		0.60		0.60		0.60
BDE-99		2.0–4.5		64.23		4.72 (3.74–5.94)		< 2.0		249.08		1.00		5.50		12.59		49.83
BDE-100		1.2		89.05		4.19 (3.51–5.00)		< 1.2		107.45		2.27		4.61		7.20		25.85
BDE-85, -155		1.2		16.06		NA		< 1.2		10.49		0.60		0.60		0.60		4.58
BDE-153		1.2		96.35		5.93 (5.09–6.92)		< 1.2		67.55		3.82		5.65		9.82		32.33
BDE-154		1.2		48.18		N/A		< 1.2		52.89		0.60		0.60		2.40		7.59
∑PBDEs*a*								3.59		693.95		20.02		36.56		64.58		228.16
Phenolic metabolites (*n* = 57)*b*																		
246 TBP		1.4–2.5		38.18		NA		< 1.4		150.74		1.65		2.43		15.14		119.71
4’-OH-BDE-49		0.01–0.03		71.93		0.11 (0.07–0.16)		< 0.01		3.92		0.02		0.12		0.27		2.32
6-OH-BDE-47		0.01–0.03		66.67		0.17 (0.10–0.29)		< 0.01		10.79		0.02		0.19		0.57		5.82
∑OH-BDEs*c*								< 0.03		13.28		0.10		0.33		0.96		6.99
Abbreviations: Max, maximum; Min, minimum; NA, not available (detection frequency was < 50%). **a**∑PBDEs variable includes BDE‑47, BDE‑99, BDE‑100, and BDE‑153 because each of these had > 50% detection rates. In creating this summed variable of the four PBDEs, observations that were below detection limit were included as one-half MDL. **b**For 246 TBP, total *n* = 55. **c**∑OH‑BDEs includes 4´-OH-BDE‑49 and 6-OH-BDE‑47 because each of these had > 50% detection rates.																		

*PBDEs.* PBDE data are available for 137 of the 140 women enrolled (Table 2). Eight of the 27 PBDE congeners measured were found to be above MDLs in the serum samples analyzed. Attempts were made to measure BDE-209; however, laboratory blank contamination with BDE-209 resulted in all values below the MDL. Total PBDEs (defined as the sum of BDEs 47, 99, 100, and 153) were log-normally distributed; however, individual PBDEs were not normal or log-normally distributed. At least one of the BDE congeners was detected in all samples, and total PBDE concentrations ranged from 3.6 to 694 ng/g lipid (Table 2).

The most abundant congener was 2,2´,4,4´-tetrabromodiphenyl ether (BDE-47), which ranged in concentration from < 2.0 to 297 ng/g lipid, with a geometric mean value of 16.5 ng/g lipid. BDE-47 contributed 50% of the total PBDE burden, on average. The second most abundant BDE congener was 2,2´,4,4´,5,5´-hexabromodiphenyl ether (BDE-153) and the most commonly detected (~ 96% of samples), with concentrations ranging from < 1.2 to 67.6 ng/g lipid. BDE-153 was approximately 20% of the total PBDE burden, on average, although in one individual serum sample BDE-153 was the only congener detected. On average, 2,2´,4,4´,5-pentabromodiphenyl ether (BDE-99) and 2,2´,4,4´,6-pentabromodiphenyl ether (BDE-100) represented 17% and 14% of the total PBDE burden, respectively.

*HBCD and phenolic metabolites.* A subset of 57 samples was further analyzed for isomers of HBCD (alpha, beta, and gamma), 246 TBP, and four different hydroxylated PBDE congeners by liquid chromatography tandem mass spectrometry. HBCD was < 0.17 ng/g lipid in all serum extracts; however, α-HBCD was detected (0.18–0.21 ng/g lipid) in the standard reference material (SRM 1958; National Institute of Standards and Technology, Gaithersburg, MD) used for quality assurance/quality control, demonstrating that the method did recover HBCD. The measured concentration in SRM 1958 ranged from 64 to 76% of the reported value (Keller et al. 2010). 6´-OH-BDE-49 and 6-OH-BDE-99 were not detected in any samples (MDL = 0.035 ng/g lipid). 4´-OH-BDE-49 and 6-OH-BDE-47 were detected in 72% and 67% of the samples, respectively. 246 TBP was detected at the highest concentrations (< 1.4–151 ng/g lipid); however, values were above MDL in only 38% of the samples. Total OH-BDE levels (4´-OH-BDE-49 plus 6-OH-BDE-47) ranged from < 0.03 to 13.3 ng/g lipid, with a geometric mean value of 0.28 ng/g lipid.

**Figure 1:**
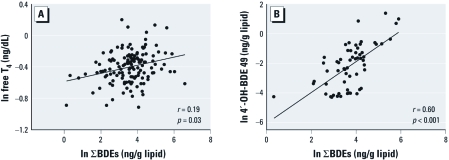
Correlations observed between PBDEs and FT_4_ (*A*), and between PBDEs and OH-BDE-49 (*B*), in serum.

*Associations between PBDEs, metabolites, and thyroid hormones.* The PBDEs were all highly correlated (*r* = 0.39–0.95) (Table 3). Total PBDEs and individual BDE congeners were highly correlated with 4´-OH-BDE-49 (*r =* 0.38–0.62; Figure 1) and 6-OH-BDE-47 (*r =* 0.29–0.40). 4´-OH-BDE-49 and 6-OH-BDE-47 were also highly correlated (*r =* 0.62, *p <* 0.0001).

**Table 3:** Spearman rank correlation matrix for PBDEs (nanograms per gram lipid) and thyroid hormones with frequency of detection > 50%.

Variable	BDE-47	BDE-100	BDE-99	BDE-153	∑BDEs	4’-OH-BDE-49	6-OH-BDE-47	∑OH- BDE
PBDEs*a*																
BDE-47																
BDE-100		0.80^##^														
BDE-99		0.80^##^		0.58^##^												
BDE-153		0.52^##^		0.67^##^		0.39^##^										
∑BDEs		0.95^##^		0.85^##^		0.85^##^		0.65^##^								
4’-OH-BDE-49		0.62^##^		0.47^#^		0.56^##^		0.38**		0.60^##^						
6-OH-BDE-47		0.35**		0.44^#^		0.29*		0.43^#^		0.40**		0.62^##^				
∑OH-BDE		0.46^#^		0.49^##^		0.39**		0.46^#^		0.49^##^		0.82^##^		0.93^##^		
Thyroid hormones*b*																
TSH		0.16		0.06		0.14		–0.02		0.11		0.17		0.02		0.07
TT_4_		0.20*		0.20*		0.18*		0.10		0.21**		0.17		0.12		0.18
FT_4_		0.19*		0.09		0.17*		0.16		0.19*		0.18		0.05		0.08
TT_3_		0.10		0.03		0.03		0.07		0.07		–0.24		–0.09		–0.16
FT_3_		0.07		0.002		0.01		–0.07		0.04		–0.05		0.01		0.03
**a**Total *n* for each of the pairwise correlations with BDE-47, BDE-100, BDE-99, BDE-153, and ΣBDEs is 137. For the phenolic metabolites, including 4’-OH-BDE-49, 6-OH-BDE-47, and ΣOH-BDEs, any pairwise correlation has a total *n* of 57. **b**When a pairwise correlation includes a thyroid hormone and any of the non–OH-BDEs, total *n* is 136. With the thyroid hormones and phenolic metabolites, total *n* is 56. *p < 0.05. **p < 0.01. ^#^*p* < 0.001. ^##^*p *< 0.0001.																

None of the correlations between TSH and PBDEs were significant (Table 3). TT_4_ was correlated with most of the PBDEs (*r =* 0.18–0.21 and *p <* 0.05 for BDEs 47, 100, 99, and ΣBDEs) but not with BDE-153 (*r =* 0.10). Although TT_4_ was not significantly associated with either of the metabolites, the correlation coefficients were similar in magnitude (*r =* 0.12–0.17) to the PBDEs. FT_4_ was significantly associated with BDE-47, BDE-99, and ΣBDEs (*r =* 0.17–0.19, *p <* 0.05; Figure 1) and was marginally associated with BDE-153 (*r =* 0.16, *p* = 0.06). FT_4_ was not associated with BDE-100 nor with the metabolites. An inverse association (*r =* –0.24) between TT_3_ and 4´-OH-BDE-49 was suggestive (*p =* 0.08), but nothing was significantly associated with TT_3_ or FT_3_ in linear regression analysis.

Results of the multivariable model (Table 4) show that TT_4_ is significantly and positively associated with BDE-47, BDE-99, BDE-100, and ΣBDEs (*r =* 0.32–0.50, *p <* 0.05), but was not significantly associated with BDE 153. Logged FT_4_ is significantly and positively associated with BDE 47, BDE-153, and ΣBDE (*r =* 0.05, *p <* 0.05) but was not significantly associated with BDE-99 or BDE-100.

**Table 4:** Multiple linear regression with TT_4_ and FT_4_ and BDEs and total BDE, controlling for maternal characteristics [β (95% CI)].

Explanatory variables													
Dependent variable	ln BDE-47		ln BDE-99		ln BDE-100		ln BDE-153		ln ∑BDEs	
Thyroid hormone (*n* = 136)															
TT_4_		0.42	(0.05 to 0.78)*		0.32	(0.02 to 0.63)*		0.41	(0.003 to 0.82)*		0.12	(–0.35 to 0.58)		0.50	(0.06 to 0.94)*
ln FT_4_		0.05	(0.01 to 0.08)**		0.02	(–0.009 to 0.05)		0.02	(–0.02 to 0.06)		0.05*	(0.006 to 0.09)*		0.05	(0.01 to 0.09)*
ln TSH		0.07	(–0.02 to 0.16)		0.04	(–0.04 to 0.11)		0.01	(–0.09 to 0.11)		0.03	(–0.08 to 0.14)		0.06	(–0.04 to 0.17)
ln TT_3_		0.04	(–0.01 to 0.08)		0.01	(–0.03 to 0.05)		0.001	(–0.05 to 0.05)		0.01	(–0.04 to 0.07)		0.02	(–0.03 to 0.08)
ln FT_3_		0.01	(–0.01 to 0.03)		0.003	(–0.01 to 0.02)		0.0004	(–0.02 to 0.02)		–0.02	(–0.04 to 0.01)		0.01	(–0.02 to 0.04)
**a**These models report the individual BDE congener–thyroid hormone association after controlling for smoking status, maternal race, age, gestational age at blood draw, and parity. *p < 0.05. **p < 0.01.															

These results hold equally well in logistic regression models (results not shown). In these models, PBDEs and OH-BDEs were log base 2 transformed so that their estimated odds ratios (ORs) are interpreted as the change in odds associated with a 2-fold increase in the particular PBDE or OH-BDE. Adjusted ordinal logistic regression model ORs for normal versus low or high versus normal TT_4_ levels with a 2-fold increase in exposure were 1.42 [95% confidence interval (CI), 1.08–1.86, *p =* 0.01] for BDE-47; 1.37 (95% CI, 1.01–1.84, *p =* 0.04) for BDE-100; 1.25 (95% CI, 1.00–1.57, *p =* 0.05) for BDE-99; and 1.45 (95% CI, 1.04–2.01, *p* = 0.03) for ΣBDEs. Likelihood ratio tests of the proportionality of odds assumption did not find evidence to reject the null hypothesis that the odds were the same across outcome categories. For each 2-fold increase in BDE-47, the odds of having low FT_4_ decreased by 40% (OR = 0.60; 95% CI, 0.39–0.93, *p =* 0.02) and by 49% for ΣBDEs (OR = 0.51; 95% CI, 0.27–0.97, *p =* 0.04). Although PBDEs (Table 4) and OH-BDEs (data not shown) were not associated with TT_3_ modeled as a continuous variable, high TT_3_ levels (178 ng/dL) were positively associated with BDE-47 (OR = 1.30; 95% CI, 1.00–1.69, *p =* 0.04) and inversely associated with 4´-OH-BDE-49 (OR = 0.51; 95% CI, 0.30–0.86, *p =* 0.01) and ΣOH-BDEs (OR = 0.72; 95% CI, 0.48–1.07, *p =* 0.10).

## Discussion

The PBDE levels and congener distributions in this pregnancy cohort were similar to levels reported among the general U.S. population in the 2003–2004 National Health and Nutrition Examination Survey (NHANES) (Sjodin et al. 2008). This similarity was unexpected, because PBDEs have been phased out from use since 2004, and there was a 4- to 7-year time gap between the sample collections for the two cohorts. One might expect to see declining levels of PBDEs in human tissues over the preceding 4–7 years. Our data suggest that levels are stable; however, some caveats must be noted with this comparison. The NHANES study observed higher PBDE levels in non-Hispanic blacks relative to non-Hispanic whites, and decreasing PBDE levels with age; therefore, levels may be comparable because our cohort was composed primarily of non-Hispanic black females ages 18–22 years. Geometric mean BDE-47 levels in NHANES (2003–2004) for 12- to 19-year-olds (28.2 ng/g lipid), 20- to 39-year-olds (21.5 ng/g lipid), and non-Hispanic blacks (24.3 ng/g lipid) were higher than the geometric mean level measured for our cohort (16.5 ng/g lipid). Thus, our results are consistent with decreasing PBDE levels over time within race and age groups.

Several phenolic metabolites were also identified in a subset of these samples (Table 2). Previous studies have observed these phenolic compounds in both human tissues and in laboratory animals exposed to PBDE mixtures, suggesting they are in fact metabolites of PBDEs (Athanasiadou et al. 2008; Hakk and Letcher 2003; Qiu et al. 2007, 2009; Wan et al. 2010). Qiu et al. (2009) reported mean levels of 0.8, 0.3, and 0.3 ng/g lipid for 246 TBP, 4´-OH-BDE-49, and 6-OH-BDE-47, respectively, in maternal blood samples (*n* = 4) collected in 2003–2004. These levels are similar to the median levels we report here of < 2.5, 0.11, and 0.17 ng/g lipid for 246 TBP, 4´-OH-BDE-49, and 6-OH-BDE-47, respectively. Although 246 TBP is a known metabolite of PBDEs, it is also produced and used as a flame retardant in phenolic and epoxy resin itself (Andersson et al. 2006). Thus, there can be more than one source of exposure to this compound in human tissues.

All PBDEs and OH-BDEs were highly correlated, except 6-OH-BDE-47 and BDE-99. This weaker relationship may be attributable to the fact that 6-OH-BDE-47 is not a likely metabolite of BDE-99 and/or may have marine natural sources (Malmvarn et al. 2005). In addition, BDE-99 may be metabolized much more rapidly than BDE-47 and BDE-153 (Chen et al. 2006; Hakk et al. 2002; Lupton et al. 2009; Stapleton et al. 2004b, 2009).

Positive associations between PBDEs and FT_4_ and TT_4_ levels have been observed in nonpregnancy cohorts in previous studies (Meeker et al. 2009; Turyk et al. 2008). Unfortunately, we were not able to control for potential confounders [e.g., polychlorinated biphenyls (PCBs), organochlorine pesticides] in this study. However, both Turyk et al. (2008) and Chevrier et al. (2010) were able to control for these confounders, and they demonstrated that associations between PBDEs and thyroid hormones remained. In addition, PCBs have been associated with decreases in T_4_, not increases as observed in our study (Brouwer et al. 1998; Wang et al. 2005).

Positive associations between PBDEs and T_4_ observed are opposite to those typically observed in laboratory animal exposure studies, where exposed animals experience decreases in circulating T_4_ and T_3_ levels (Fernie et al. 2005; Tomy et al. 2004; Zhou et al. 2001). A previous study examining PBDEs in pregnant women found no significant associations between PBDEs and T_4_; however, the authors did observe an inverse association between PBDEs and TSH (Chevrier et al. 2010). Low TSH levels are generally associated with higher T_4_ levels via feedback mechanisms regulated by the pituitary (Hulbert 2000); thus, the results observed here and by Chevrier et al. (2010) suggest a similar trend with respect to the influence of PBDEs on thyroid hormone regulation during pregnancy. Differences in TSH associations between the two studies may be related to the fact that African-American women in general have lower TSH levels (Walker et al. 2005). Both cohorts displayed very similar PBDE levels, with geometric mean BDE-47 levels of 15.3 and 16.9 for their cohort and our cohort, respectively. However, as noted by Chevrier et al. (2010), different methods for measuring thyroid hormones may also be a factor in different trends observed. Here we used a chemiluminescent immunoassay for measuring unbound (i.e., free) thyroid hormones (which may be a limitation), whereas Chevrier et al. (2010) used an equilibrium dialysis method. Diurnal variations in thyroid hormone secretions may also be a factor.

Our findings suggest an inverse association between 4´OH-BDE-49 and TT_3_, but our analysis was limited by the small sample number (*n* = 56). Most animal exposure studies have observed significant decreases in T_4_ with exposure to PBDEs, but not T_3_. However, a large proportion of this cohort (63%) did have TT_3_ levels that were above normal levels for an average adult, which is likely linked to their late stage of pregnancy. Previous *in vitro* studies have demonstrated that hydroxylation of the PBDE congener results in significantly increased binding affinities for thyroid hormone transporters found in serum (Marchesini et al. 2008; Meerts et al. 2000). Specifically, 4´-OH-BDE-49 has been shown to have a high binding affinity to the thyroid hormone serum transporter transthyretin (Ucan-Marin et al. 2009). Thus, it may be possible that PBDE metabolites are competing for space on the thyroid hormone transporters, resulting in more free (i.e., unbound) T_4_. However, TT_4_ was also positively associated with PBDEs. An alternate explanation may be related to the effect of PBDEs and/or their metabolites on deiodinase activity. Previous *in vivo* and *in vitro* studies in fish models have suggested that deiodinase enzymes (DIs) may be metabolizing PBDEs via a dehalogenation pathway (Noyes et al. 2010; Stapleton et al. 2004a). DIs serve to metabolize T_4_ to T_3_ in peripheral tissues, supplying the main source of T_3_ that binds to thyroid nuclear receptors to activate transcriptional processes (Kohrle 1999). If DI activity was inhibited by PBDEs and/or their metabolites, it might result in an increase in circulating T_4_ levels and a decrease in T_3_ levels.

Here we have reported on the levels of PBDEs in a pregnancy cohort consisting primarily of African-American women and report on the associations between OH-BDEs and thyroid hormones. Although there are some limitations to this study (e.g., analysis during the third trimester of pregnancy, method used for measuring thyroid hormones), the results do support previous studies that have observed associations between PBDEs and thyroid hormones. PBDEs have a very long half-life in the human body, and PBDE levels measured in the third trimester are likely to reflect levels throughout pregnancy. Consequently, it is possible that PBDEs may be affecting thyroid hormone regulation throughout pregnancy, including the first trimester when the fetus relies solely on maternal thyroid hormone supply. More research is needed to determine the mechanisms by which PBDEs affect thyroid hormone regulation during pregnancy and how this, in turn, affects the development of the fetus.

## Supplemental Material

(40 KB) PDF
