# Supplementary material for: Associations between Polybrominated Diphenyl Ether (PBDE) Flame Retardants, Phenolic Metabolites, and Thyroid Hormones during Pregnancy
Source: Environ Health Perspect. 2011 Jun 29;119(10):1454–9. doi: 10.1289/ehp.1003235 (PMC3230439; doi:10.1289/ehp.1003235)
Supplement: (40 KB) PDF [file ehp.1003235.s001.pdf]

Supplemental Materials

Associations Between Polybrominated Diphenyl Ether (PBDE) Flame Retardants, Phenolic Metabolites, and Thyroid Hormones During Pregnancy

Supporting Information

Heather M. Stapleton*[1], Sarah Eagle[1], Rebecca Anthopolos[1], Amy Wolkin[2], and Marie Lynn Miranda[1,3],

1. Nicholas School of the Environment, Duke University, Durham, NC, USA
2. National Center for Environmental Health, Centers for Disease Control and Prevention, Atlanta, GA, USA
3. Department of Pediatrics, Duke University, Durham, NC, USA

Address correspondence to:
Heather Stapleton, PhD
Nicholas School of the Environment
Duke University
LSRC, Box 90328
Durham, NC 27708
Phone: 919-613-8717
Fax: 919-684-8741
Email: heather.stapleton@duke.edu

Chemicals. All solvents used for the analysis were HPLC-grade or better. A fluorinated BDE standard, 4'-2,3',4,6-tetrabromodipheyl ether (FBDE-69) (Chiron Inc., Trondheim, Norway) was used as an internal and recovery standard. The recovery of FBDE-69 was assessed by addition of ^{13}C labeled 2,2',3,4,5,5'-hexachlorinated diphenyl ether (Cambridge Isotope Laboratories, Andover, MA). PBDE calibration solutions were prepared from neat standards purchased from Accustandard Inc. (New Haven, CT). Standard solutions of alpha-, beta- and gamma hexabromocyclododecane (HBCD), 2,4,6-tribromophenol, and ^{13}C labeled 6-hydryoxy-2,2',4,4'-tetrabromodiphenyl ether (^{13}C 6-OH-BDE 47) were purchased from Wellington Laboratories (Guelph, Ontario). Standard solutions of 4'-hydroxy-2,2',4,5'-tetrabromodiphenyl ether (4'-OH-BDE 49), 6'-hydroxy-2,2',4,5'-tetrabromodiphenyl ether (6'-OH-BDE 49), 6-hydroxy-2,2',4,4'-tetrabromodiphenyl ether (6-OH-BDE 47) and 6-hydroxy-2,2',4,4',5-pentabromodiphenyl ether (6-OH-BDE 99) were purchased from Cambridge Isotope Laboratories (Andover, MA).

Sample Extraction Serum samples (approximately 3-5 grams) were extracted using a previously published method (Stapleton et al. 2008). Briefly, serum samples were first spiked with FBDE-69 (25 ng) and then treated with hydrochloric acid to denature the proteins. Serum was extracted by liquid-liquid extraction and cleaned using acid silica. The final extract was reduced in volume to 0.5 mL, transferred to a GC auto-sampler vial and spiked with 25 ng of the recovery standard, ^{13}C CDE-141.

Sample Analysis Analytes were separated by liquid chromatography (Agilent 1200; Agilent, Santa Clara, CA) on a Zorbax Eclipse XBD-C18 column (1.8 µm, 4.6 x 50 mm; Agilent) under isocratic conditions of 90% methanol and 10% water at 0.4 mL min^{-1}. Injection volume was 15 µL. Compounds were detected by multiple reaction monitoring (MRM) using tandem mass spectrometry with negative electrospray ionization (Agilent 6410B triple quadrupole spectrometer, Santa Clara, CA). Source parameters were as follows: gas temperature 350°C, gas flow 10 L min $^{-1}$, nebulizer pressure 40 psi, and capillary voltage

4000 V(-). Internal standards included ^{13}C αHBCD for HBCD isomers and ^{13}C OH-BDE 47 for all OH-BDEs and 246 TBP. The different analytes measured by LC/MSMS and their MRM parameters are: ^{13}C α-HBCD (652.7 > 78.9), α,β,γ-HBCD (642.6>78.9, 640.6 > 78.9), ^{13}C 6-OH-BDE 47 (512.8 > 78.9), 4'-OH-BDE 49 (500.8> 266), 6-OH-BDE 47 and 6-OH-BDE 49 (500.8 > 78.9), 6-OH-BDE 99 (520.9 > 78.9), and 245 TBP (328.8 > 78.9, 330.8 > 78.9). Fragmentor voltages were set at 80 V for HBCD and OH-BDEs and 120 V for 246 TBP. Collision energy was set at 15 V for HBCD isomers, 20 V for OH-BDEs, and 40 V for 245 TBP.

Quality Control Minor laboratory contamination with BDE-47(0.23-0.28 ng), BDE-99 (0.10-0.13ng) and 2,4,6-tribromophenol(0.18 to 0.76 ng) was observed. All samples were blank subtracted using the average blank measurement for these three congeners on a batch by batch basis. Method detection limits (MDL) were calculated at 3 times the standard deviation of the blanks normalized to the serum sample mass and lipid content. The MDLs for the remaining congeners were determined by calculating the mass equivalent to three times the signal noise and normalizing the mass to the serum sample mass and lipid content. Recovery of the internal standard, FBDE-69 averaged 78 ± 12%, and recovery of the BDE congeners in SRM 1958 ranged from 90 to 109% for PBDEs and 64-76% for alpha-HBCD. Recovery of the four OH-BDE congeners was assessed by spiking 5 ng of each congener into 5 mL of bovine serum. Recoveries for the four OH-BDE congeners ranged from 59 to 76%.

References

Stapleton HM, Sjodin A, Jones RS, Niehoser S, Zhang Y, Patterson DG. 2008. Serum levels of polyhrominated diphenyl ethers (PBDEs) in foam recyclers and carpet installers working in the united states. Environmental Science & Technology 42(9): 3453-3458.
